# Identifying stakeholders and analyzing their concerns about African swine fever control in wild boar

**DOI:** 10.3389/fvets.2025.1602027

**Published:** 2025-07-29

**Authors:** Janine Miesch, Jana Schulz, Barbara Thür, Katja Schulz, Annika Frahsa, Salome Dürr

**Affiliations:** ^1^Vetsuisse Faculty, Veterinary Public Health Institute, University of Bern, Bern, Switzerland; ^2^Institute of Epidemiology, Friedrich-Loeffler-Institute, Federal Research Institute for Animal Health, Greifswald, Germany; ^3^Cantonal Veterinarian, Office for Consumer Protection and Veterinary Service, Aargau, Switzerland; ^4^Institute of Social and Preventive Medicine, Faculty of Medicine, University of Bern, Bern, Switzerland

**Keywords:** ASF, wild boar management, stakeholder analysis, participatory research, veterinary public health, wildlife disease control, crisis management

## Abstract

African swine fever (ASF) is approaching Switzerland as it continues to spread across Europe. This viral disease affects porcine species, leading to severe economic losses when reaching the domestic pig sector. Controlling ASF in wild boar populations is complex and requires coordination among diverse stakeholders with varying roles and interests. We used a participatory approach in data generation (including desk research, qualitative interviews, focus group discussions, and workshops) and applied reflexive thematic analysis to systematically identify relevant actors and assess their concerns, guided by the Mendelow Power-Interest Grid for stakeholder mapping. Results reveal a broad spectrum of stakeholders, including federal and cantonal-level authorities, the private industry sector, non-governmental organizations (NGOs) and private individuals, as well as academic and diagnostic institutions. Stakeholder mapping underscores the central role of federal and cantonal authorities in ASF control and demonstrates the hunting sector’s dual position as both being impacted by and being actively involved in control efforts. Stakeholders’ concerns fall into five key areas: economic risk, material shortages, legal frameworks and bureaucratic obstacles, challenges in communication and coordination, and animal welfare and environmental issues. Findings emphasize the need for improved governance, clearer guidelines, and stronger coordination among federal and cantonal authorities. The research demonstrates the value of participatory approaches for disease management by enhancing collaboration, identifying critical gaps, and strengthening preparedness and response efforts, on the example of ASF in Switzerland.

## 1 Introduction

African swine fever (ASF) poses a growing threat to European countries since it continues to be spreading westwards into central Europe (1). The severe hemorrhagic contagious viral disease affects domestic pigs and Eurasian wild boars (*Sus scrofa*) and has a high lethality in infected animals. ASF incursions can negatively impact pork production, exports, and pig inventories, especially when outbreaks are widespread or affect domestic pigs (2). Therefore, prevention, early detection and control of the disease in wildlife, with the goal of disease freedom, are primary objectives for most European countries (3, 4).

In Switzerland, ASF has not been reported yet; however, the country borders affected countries, such as Germany and Italy (1). As a federal state composed of 26 cantons, each with its own constitution and considerable autonomy, responsibilities in animal disease control are shared between national and cantonal levels. The federal government defines overall strategies and provides coordination, while cantonal veterinary authorities are tasked with implementing and adapting these measures locally. Switzerland has implemented an early detection program for ASF. Awareness campaigns targeting hunters, pig farmers, and travelers aim to promote early detection and to minimize the probability of disease introduction through laboratory testing of wild boars found dead and sick hunted, improved pig farm biosecurity measures, and targeted banning of pork product imports (5). Switzerland has also developed a contingency plan in case of ASF incursion into the wild boar population, which includes the following three main approaches: establishing quiet zones to minimize human disturbance and prevent the infection’s spread through wild boar movement; locating and disposing of wild boar carcasses to reduce environmental contamination with ASF-virus and the probability of new infections; and, depending on the affected area, implementing comprehensive culling or targeted population reduction (6).

Managing wildlife diseases requires the coordination of diverse stakeholders with varying interests and expectations. A systematic identification of these stakeholders, coupled with an understanding of their perspectives, is critical for successful control. A participatory approach ensures that stakeholder concerns are addressed, enabling policymakers to communicate effectively and refine regulatory frameworks, and finally enhance the effectiveness of control strategies. Participatory research encompasses various research designs, methodologies, and frameworks that systematically involve collaboration with entities directly impacted by the investigated topic, with the aim of driving action or facilitating change (7). It also emphasizes the co-construction of findings by fostering partnerships between researchers and stakeholders, including community members and individuals with firsthand knowledge and lived experience (8).

Various methods have already been used to capture stakeholder perspectives in the fight against ASF in wild boar. For example, a study in Lithuania used focus groups and semi-structured interviews to gather Lithuanian hunters’ knowledge and perceptions of ASF control and surveillance, aiming to incorporate their insights for more effective disease control measures (9). Urner et al. also employed participatory methods to assess the acceptance of ASF control measures among Latvian hunters and explored strategies to enhance their motivation for passive surveillance (10). The research conducted by Jori et al. employed the World Café method, a structured but informal format involving rotating small-group discussions, to collect expert opinions on the effectiveness and acceptance of different ASF control strategies in European wild boar populations, yielding important recommendations for collaborative ASF control programs (11).

The first step in effectively conducting participatory research is a structured identification of stakeholders through stakeholder analysis. This includes systematic identification and mapping of potentially relevant stakeholders. Stakeholder analysis employs collection and analysis of qualitative information to determine whose interests should be considered in the development or implementation of a policy or program. Within this project, we conducted a stakeholder analysis in Switzerland to identify relevant stakeholders using a participatory research methodology. The participatory process also ensured the selection of appropriate representatives who are recognized by each stakeholder group. In addition, we captured the stakeholders’ concerns and considerations related to ASF control in wild boar during stakeholder workshops and analyzed them systematically to identify key themes and patterns. The results of this project are expected to serve as a foundation for a successful ASF control program in wild boar in Switzerland.

## 2 Methods

### 2.1 Transdisciplinary research approach

On the continuum of participatory approaches, this work is positioned at the level of transdisciplinary research that integrates expertise across disciplines and engages stakeholders from non-academic backgrounds. The study employs a critical realist perspective, which assumes that a reality exists independently of our perceptions, to address a complex, multi-dimensional problem through knowledge exchange and co-construction of ASF control strategies in wild boar (12–15).

This project operates at the “placation” level on Arnstein’s Ladder of Citizen Participation (score 5 of 8), a framework that describes different levels of public involvement in decision-making. At this level, stakeholders are given the opportunity to express their views and provide advice, but the power to make final decisions remains entirely with public authorities (16). In our case, stakeholders were involved through interviews, discussions, and workshops to contribute their perspectives and concerns to ASF control planning.

### 2.2 Stakeholder identification

The first step consisted of stakeholder identification in alignment with the Reporting Items for Stakeholder Analysis (RISA) tool by L. Franco-Trigo et al. (Supplementary Table 1) (17). The RISA tool provides a structured guideline to enhance the quality and transparency of stakeholder analysis reporting. Stakeholders were defined as a person, group, or organization with an interest or concern in the topic under investigation and who can affect or be affected by its actions, objectives, or policies.

A preliminary list of stakeholders was first identified through a review of peer-reviewed literature and grey literature, including technical guidelines, official reports, and applied field studies from ASF control efforts in other European countries, accessed through targeted searches of institutional sources and relevant online repositories (6, 11, 18, 19). This also informed the selection of initial interview partners. Thereafter, we conducted 11 semi-structured interviews with selected experts in the field to confirm the list and identify missing stakeholders. All interviewees were based in Switzerland and selected for their relevant institutional roles. Further experts were added through interviewee recommendations. The interviewees included an ASF-responsible officer from the Federal Food Safety and Veterinary Office (FSVO), two cantonal chief veterinarians, a lead veterinary official, a representative from the Forest, Wildlife, and Landscape Association, a responsible officer from the Federal Office for the Environment (FOEN), a representative from the Federal Office for Agriculture (FOAG), a wild boar research specialist, the leading officer of a cantonal hunting office, an expert from the Swiss Federal Institute for Forest, Snow and Landscape Research (WSL) and a specialist from the Center for Public Management at the University of Bern. These interviews also gathered data essential for subsequent categorization of stakeholder groups and mapping efforts. We carried out the interviews between August and November 2023. Interview data were recorded through note-taking and later discussed within the research team to extract relevant stakeholder information for categorization and mapping.

### 2.3 Stakeholder mapping and categorization

We used three approaches to map the identified stakeholders. First, we applied the Mendelow Power-Interest Grid for mapping and categorization, classifying stakeholders according to their power levels versus interest related to ASF control in wild boar in Switzerland (20). The Mendelow Grid is a framework that categorizes stakeholders based on their level of influence (or, power) and interest to determine appropriate engagement strategies. We categorized stakeholders into four types: Strategic Partners with high power but low interest, providing oversight and resources; Active Collaborators with both high power and interest, shaping strategy and implementation; Informed Advocates with high interest but low power, offering insights and supporting communication; and Peripheral Observers with low power and interest, requiring minimal engagement. The initial placement of stakeholders in these four types was based on the review of literature and documents from ASF control in other European countries and contextual knowledge. This categorization was then refined through the 11 semi-structured expert interviews. In each interview, the draft grid was shown to the interviewees, who could suggest reassignments based on their experience. Suggested changes were incorporated and shown again in subsequent interviews for further feedback. This iterative process allowed validation and adjustment of the mapping based on expert input. Power was defined as a stakeholder’s authority or capacity to shape ASF control decisions and outcomes, while interest referred to the level of concern, involvement, or perceived relevance of ASF control to the stakeholder’s responsibilities or goals, including economic, ecological, or operational considerations. The analysis was conducted by a senior researcher with extensive experience in transdisciplinary and participatory research, together with a junior researcher in veterinary public health. Their combined expertise informed the interpretation of stakeholder power and interest in the Swiss context.

We further categorized stakeholders based on their roles in ASF control, distinguishing between those primarily affected by control measures, those actively influencing or implementing them, and those with both characteristics (21). Stakeholders categorized as affected are those whose regular activities or operational fields are significantly impacted, typically constrained, by the measures. Conversely, stakeholders categorized as affecting are the primary implementers or influencers of the measures. This classification was developed through internal discussions within the research team, drawing on insights gained from the expert interviews, and guided by the stakeholders’ institutional responsibilities and observed positions within the ASF response landscape. To further specify stakeholder roles, we indicated the approximate level of involvement in ASF control activities as “most, moderate, or least,” based on their known mandates, expected responsibilities, and information provided during expert interviews.

We finally used another approach to categorize the stakeholders as “Movers,” who are committed to supporting and actively contributing to the implementation of control measures, form strategic alliances and maintain close relationships with one another to advance the shared goal; “Blockers,” who oppose may attempt to hinder the control measures, often due to conflicting values or their own interests being negatively impacted; and “Floaters,” who occupy an intermediate position, neither fully supporting nor obstructing the process, and who passively implement the measures without strong opinions which may shift depending on the communication and engagement strategies employed. This classification is based on their stance toward ASF control measures for wild boar, i.e., whether proactive, resistant, or neutral, respectively (22). This analysis positions stakeholders based on their interest and alignment with measures to combat ASF in wild boar populations. Furthermore, this framework provides a nuanced understanding of stakeholder dynamics, offering insights into potential areas of collaboration and conflict. As with the previous framework, this categorization emerged through internal discussions within the research team, drawing on recurring themes from the interviews to synthesize stakeholder attitudes and expected behaviors in the context of ASF control.

### 2.4 Focus group discussions

Subsequently, we conducted four online focus group discussions in January 2024. The power-interest mapping was utilized to identify participants for the focus group discussions. Representatives within the categories active collaborators, informed advocates, and strategic partners were invited to participate in focus group discussions. Ultimately, each of these stakeholders within the mentioned categories had at least one representative present during the focus group discussions. The only exception was the political stakeholders from the category of strategic partners, such as members of parliament involved in ASF-related motions, for whom we were not able to identify a participant. Stakeholders were contacted and invited via email based on their categorization in the stakeholder mapping. For each focus group, 10–20 individuals were invited. We conducted the focus group discussions with sector-specific stakeholder groups identified through the stakeholder mapping: pig industry and agriculture (participants *n* = 10), hunting and forestry (*n* = 12), forest users and animal/environmental protection (*n* = 6), and crisis management (*n* = 8). In the pig industry and agriculture group, participants included representatives from the national pig meat association, livestock health services, pig health service, a company representing Swiss pig producers and international genetics clients, the national farmers union, three additional regional agricultural associations, cantonal agricultural offices, and the federal office for agriculture. The hunting and forestry group included three cantonal hunter associations, two cantonal hunting offices, the Swiss forestry association, three cantonal forest offices, the professional association of forestry personnel, and two associations of forest owners. The forest users and animal/environmental protection group included the national dog association, a representative of a national forest stewardship association, two animal protection organizations, and two environmental protection organizations. The crisis management group included representatives from the Swiss army, the federal civil protection agency, the Swiss fire service, traffic coordination authorities, cantonal disaster response and emergency planning units, and cantonal representatives of civil protection. No participants attended more than one focus group. All discussions were conducted by two moderators: a senior researcher in veterinary public health with extensive experience in transdisciplinary approaches, and a junior researcher in veterinary public health sciences. Data collection was conducted through note-taking. The objectives of the focus group discussions were threefold: (a) to introduce the project and the upcoming participatory research workshops, (b) to assess the comprehensiveness and accuracy of the stakeholder analysis (identification and mapping), and (c) to facilitate a collaborative process in which each stakeholder group selected their recognized and interested representatives for the project’s workshops. Each session lasted approximately 60–70 min, with about 30 min dedicated to project introduction, 20 min to the assessment of comprehensiveness and accuracy, and 10–20 min to the selection of representatives. The second and third parts of the discussions were guided using open-ended prompts to encourage participant input, thus following an open, unstructured approach.

### 2.5 Workshops

We conducted a series of four workshops with two separately formed participant cohorts (cohort 1: *n* = 16, cohort 2: *n* = 16), resulting in a total of eight workshops. Participation across the eight workshops was consistent, with most individuals attending regularly. Each cohort included representatives from all selected stakeholder groups rather than being composed of specific sectors. The first workshops were conducted in March 2024. All workshops were held in person and followed a semi-structured format designed to facilitate dialogue and reflection. These sessions provided a platform for participants to engage in a discussion of concerns, questions, and uncertainties pertaining to the control of ASF in wild boar populations. During the plenary sessions, the participants were asked to voice the concerns and considerations of the stakeholder group they represented. These concerns were recorded on sticky notes to ensure a comprehensive capture of stakeholder perspectives. The sticky notes were grouped, and their content summarized by one of the workshop facilitators, a senior researcher in veterinary public health with extensive experience in transdisciplinary approaches. The summarized content was then discussed in the plenum. The workshop proceedings were documented in detail by designated note-takers, all veterinarians with specialization in epidemiology.

### 2.6 Reflexive thematic analysis

We analyzed the statements expressed by the workshop participants with a reflexive thematic approach, following Braun and Clarke (23), which involves both describing and interpreting the data. The analysis was conducted on the combined data from the two workshop cohorts, consisting of workshop documentation and notes. Data from the earlier research activities (interviews and focus groups) were also documented through note-taking, however, the thematic analysis of concerns and considerations was based exclusively on the data collected during the structured workshop exercise. We familiarized ourselves with the data through repeated readings to gain a comprehensive understanding and note initial insights. Subsequently, we generated initial codes through a systematic examination, identifying relevant features in a flexible, non-hierarchical manner (Supplementary Table 2). Initial coding and theme generation were conducted by one researcher. Preliminary themes were then reviewed and refined by two additional members of the research team, who were also familiar with the data and the coding framework. Any disagreements were resolved through discussion until consensus was reached. We then organized the codes into potential themes, a creative and interpretive phase to identify patterns within the data. We reviewed preliminary themes and refined them to ensure they accurately represented the data, with some themes revised or merged. Finally, the themes were clearly defined and named (Supplementary Table 3). Themes were not formally presented back to participants for review; however, the final results, including the five overarching themes, were shared in summary form.

### 2.7 Ethical considerations

The jurisdictional review of the Kanton Bern Ethics Committee confirmed that the project fell outside the Swiss Human Research Act (Art. 2, Paragraph 1), and therefore granted a waiver for ethical approval (BASEC number: Req-2023-01233, received on 13/10/2023). We did not offer financial compensation for participation but compensated for travel costs and provided meals. Participants had the option to withdraw at any time.

## 3 Results

### 3.1 Stakeholder identification

After the exploration of the literature and the 11 expert interviews, a large range of potentially relevant stakeholders was identified, including governmental entities (federal and cantonal level), private industry, non-governmental institutions, forest users, and research and diagnostic institutions. We categorized stakeholders by their roles and jurisdiction, grouping them based on their functions and operational levels (Table 1).

**Table 1:** Overview of stakeholder groups and representative institutions or actors involved in African swine fever (ASF) control in Switzerland.

Stakeholder group	Institutions/actors
Federal Level Authority – governmental	Federal Food Safety and Veterinary Office (FSVO)
	Federal Office for Agriculture (FOAG)
	Federal Office for the Environment (FOEN)
	Federal Office for Civil Protection (FOCP)
	Federal Office for Civilian Service (FOCS)
	Federal Roads Office (FEDRO)
	Swiss Armed Forces – Competence Center for Veterinary Services and Army Animals
	Border Veterinary Service
	Umbrella organizations: KWL, VSKT, LDK
Cantonal Level Authority – governmental	Cantonal veterinary offices
	Cantonal hunting authorities and game wardens
	Cantonal forestry and environmental offices
	Cantonal agricultural offices
	Cantonal civil protection organizations
Private Industry Sector: Pig and pork production, agriculture, pharmaceutical industry, tourism, and media	Pig farmers and pork producers
	Swiss Association for Pig Breeding and Production (Suisseporc)
	Animal health services (NTGS as the umbrella organization, with SUISAG and Qualiporc providing health programs)
	Swiss Meat Association (Proviande)
	Agricultural producers
	Feed industry (Swiss Association of Feed Manufacturers, Landi, UFA AG)
	Veterinary pharmaceutical industry (Zoetis Switzerland GmbH, Elanco Animal Health, Boehringer Ingelheim Animal Health Switzerland, Virbac Switzerland)
	Forestry professionals (Swiss Association of Forestry Personnel, Swiss Forestry Society)
	Veterinary practitioners
	Slaughterhouses
	Transport and rendering companies (GZM Centravo und TMF Bazenheid)
	Tourism agencies
	Media
Non-Governmental Organizations (NGOs) and private actors	Hunters and hunting associations (Berner Jägerverband, JagdAargau, JagdThurgau, JagdSchweiz)
	Forest owners (private and public owners, WaldSchweiz)
	Animal protection organizations (Schweizer Tierschutz, ProWildtierschutz)
	Environmental organizations (Swissrangers, Pro Natura)
	Search and detection dog units and associations (ASP-Spürhunde Schweiz)
	Drone operators
	Political stakeholders and government representatives
	Private forest users
	Tourists and travelers
	Volunteers for ASF control measure implementation
Academic, Research, and Diagnostic Institutions – governmental	Institute of Virology and Immunology (IVI)
	Vetsuisse Faculty (Institute for Fish and Wildlife Health (FIWI), Veterinary Public Health Institute (VPHI), Swine Clinic)
	Wildlife Management Research Group (WILMA)
	Geneva School of Landscape, Engineering and Architecture (HEPIA)
	Swiss Federal Institute for Forest, Snow and Landscape Research (WSL)
	Institute for Agricultural Sciences, ETH Zurich

The table presents five stakeholder groups relevant to ASF preparedness and control, each accompanied by examples of institutions or actors contributing to related activities.

At the national level, the stakeholder group “Federal Level Authority – governmental” comprises institutions with mandates in areas such as animal health, agriculture, environment, civil protection, infrastructure, and military veterinary services. These authorities are central to the formulation of legal and strategic frameworks and provide coordination and guidance for ASF prevention and control efforts across the country. Their involvement ensures consistency between federal policy and its cantonal execution. While most national governmental organizations are federal offices responsible for policy development, national umbrella organizations such as the KWL (Association of Cantonal Forest and Wildlife Authorities), the VSKT (Association of Swiss Cantonal Veterinarians), and the LDK (Association of Agricultural Authorities) serve as coordinating bodies. These organizations facilitate collaboration among cantonal authorities, align policies, and support the implementation of national strategies while respecting the decentralized structure of the Swiss federal system. The group “Cantonal Level Authority – governmental” comprises actors responsible for regional implementation of ASF control measures across Switzerland’s federal system. These authorities operate in areas such as veterinary affairs, agriculture, environment, hunting, forestry, and civil protection. Their structures and responsibilities differ between cantons, requiring flexible coordination and adaptation of federal strategies to local contexts. For example, while in some cantons Agriculture and Nature Offices manage hunting and fishing issues separately, these responsibilities fall under the Department of Construction, Transport, and Environment in other cantons. The stakeholder group “Private Industry Sector: Pig and pork production, agriculture, pharmaceutical industry, tourism, and media” includes a broad range of private-sector actors whose operations are directly or indirectly affected by ASF control measures. This encompasses stakeholders involved in livestock production, animal health, supply chains, land use, public communication, and economic sectors. The group “Non-Governmental Organizations (NGOs) and private actors” brings together a spectrum of non-state stakeholders engaged in or impacted by ASF preparedness and response. It includes organized associations as well as individual actors who contribute specific expertise or play a role in the field. National umbrella organizations such as JagdSchweiz and WaldSchweiz play a central role in representing the interests of hunters and forest owners, respectively. The group “Academic, Research, and Diagnostic Institutions” encompasses a range of governmental institutions engaged in applied and basic research related to animal health, wildlife, agriculture, and the environment. These actors contribute expert knowledge, conduct diagnostic services, and support evidence-based policymaking.

### 3.2 Stakeholder mapping

**Figure 1:**
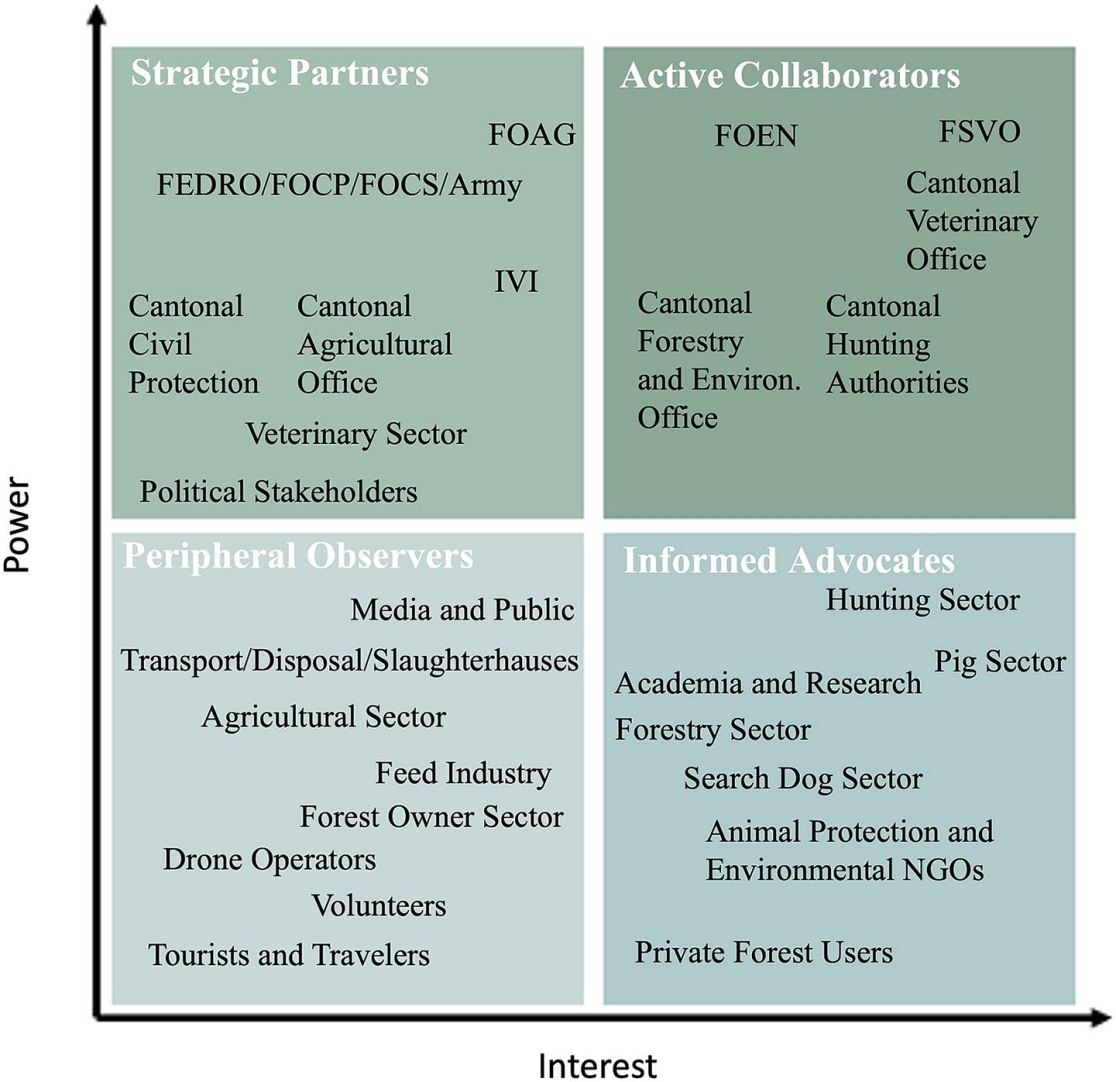
Stakeholder power-interest mapping according to Mendelow. The figure illustrates the mapping framework of stakeholders involved in the control of future African swine fever in wild boar in Switzerland based on their power and interest. Relative proximity to the axes reflects an estimated strength of stakeholder power or interest. To enhance clarity and simplify the presentation, some stakeholders were grouped under broader categories in the illustration, such as “Academia and Research,” which encompasses diverse institutions including the Vetsuisse Faculty, the Geneva School of Landscape, Engineering and Architecture, the Swiss Federal Institute for Forest, Snow and Landscape Research among others. FSVO, Federal Food Safety and Veterinary Office; FOEN, Federal Office for the Environment; IVI, Institute of Virology and Immunology; FOAG, Federal Office for Agriculture; FEDRO, Federal Roads Office; FOCP, Federal Office for Civil Protection; FOCS, Federal Office for Civilian Service.

The categorization according to Mendelow’s matrix revealed Active Collaborators as federal agencies such as the Federal Food Safety and Veterinary Office and the Federal Office for the Environment, as well as cantonal hunting authorities and cantonal forestry and environmental offices (Figure 1). The category of Strategic Partners comprised the Federal Office for Agriculture, the Federal Roads Office, the Federal Office for Civil Protection, the Federal Office for Civilian Service, the Swiss Armed Forces, and the Institute of Virology and Immunology, alongside cantonal civil protection agencies, cantonal agricultural offices, the veterinary sector, and political stakeholders such as members of parliament who advocate for the issue. The Informed Advocates group encompassed the hunting and pig sectors, academia and research institutions, the forestry sector, the search dog sector, animal protection and environmental NGOs, as well as private forest users. Finally, the Peripheral Observers category included the media and the public, transportation, disposal services, slaughterhouses, the agricultural sector, the feed industry, forest owners, drone operators, volunteers, and travelers/tourists.

Mapping the stakeholders based on their level of “being affected by” or “affecting” the ASF control measures revealed that most affected stakeholders included the pig sector, forestry sector, and private forest users, who face direct limitations or changes due to the implementation of ASF control strategies (Table 2; Supplementary Figure 1). Moderately affected stakeholders included the feed industry, agricultural sector, transportation, disposal and slaughterhouses, forest owners, as well as travelers/tourists. Affecting stakeholders, on the other hand, consisted of the federal and cantonal offices, animal protection and environmental NGOs, the media and public, academia and research institutions, political stakeholders, the search dog sector, and volunteers. These stakeholders play key roles in executing ASF control measures, shaping their feasibility, and determining their outcomes. The hunting sector was found to be uniquely positioned as both “affected” and “affecting,” given its dual role in being impacted by control measures and contributing to their implementation and success.

**Table 2:** Mapping of stakeholders in the future control of African swine fever in wild boar in Switzerland: stakeholders are categorized as movers (actively driving decisions and actions), floaters (neutral), and blockers (potential opposing forces).

Category	Mover	Floater	Blocker
Affecting most	FSVO^1^ Cantonal Veterinary Office		
Affecting moderate	Cantonal Hunting Authorities Cantonal Forestry and Environmental Office FOEN^2^ Cantonal Agricultural Office FOAG^3^ IVI^4^	Cantonal Civil Protection	
Affecting least	Search Dog Sector Academia and Research Volunteers	Political Stakeholders FEDRO^5^/FOCP^6^/ FOCS^7^/Swiss Armed Forces Veterinary Sector Media and Public	Animal Protection and Environmental NGOs
Both	Hunting Sector		
Affected most	Pig Sector	Forestry Sector	Private Forest Users
Affected moderate to least		Transport / Disposal / Slaughterhouses Feed Industry Agricultural Sector Forest Owner Sector Travelers/Tourists	

Additionally, they are classified into affecting (having an impact on control measures) and affected (impacted by ASF control efforts) groups, with varying degrees of involvement. 1: Federal Food Safety and Veterinary Office, 2: Federal Office for the Environment, 3: Federal Office for Agriculture, 4: Institute of Virology and Immunology, 5: Federal Roads Office, 6: Federal Office for Civil Protection, 7: Federal Office for Civilian Service.

The stakeholder mapping approach investigating the categories “Movers, Floaters, and Blockers” identified the Federal Food Safety and Veterinary Office, cantonal veterinary offices, the hunting sector, the pig sector, the search dog sector, cantonal hunting and forestry offices, cantonal agricultural offices, academia and research institutions, the Institute of Virology and Immunology, the Federal Office for the Environment, the Federal Office for Agriculture, and volunteers as “Movers” (Table 2; Supplementary Figure 2). The “Floaters” include political stakeholders, the Federal Roads Office, the Federal Office of Civil Service, the Army, the Federal Office for Civil Protection, the media and public, the forestry sector, the transport and disposal sector, slaughterhouses, the feed industry, the agricultural sector, forest owners, as well as travelers. The “Blockers” consist of animal protection and environmental NGOs, as well as private forest users.

### 3.3 Stakeholder concerns and considerations

Five themes were built based on codes identified throughout the reflexive thematic analysis: economic risk, material shortages, legal frameworks and bureaucratic obstacles, challenges in communication and coordination, and animal welfare and environmental issues.

#### 3.3.1 Economic risk

Concerns about the economic impacts of ASF control measures were identified, particularly on forestry, domestic pig production, and agriculture. Prominent issues included financial losses, restricted forest use, and declining meat consumption. Although outdoor pig farming is rare in Switzerland, legal restrictions in protection zones would temporarily prohibit such systems, adding to the sector’s vulnerability. Compensation for lost income due to harvest bans, work restrictions for foresters, and blocked timber use was discussed. Questions also arose about funding mechanisms and cost-sharing between federal and cantonal authorities.

#### 3.3.2 Material shortages

The availability of materials and personnel for the ASF control emerged as a critical issue, alongside the logistical challenges of implementing containment measures of wild boar population. The national planning and provision of resources, as well as timely preparation for crisis management, were highlighted.

#### 3.3.3 Legal frameworks and bureaucratic obstacles

Legal frameworks and bureaucratic hurdles were reported as major barriers, including the effect of federalism (i.e., the independent sovereignty of cantonal authorities), administrative burdens, and unclear legal responsibilities in crisis management. Stakeholders noted conflicting legal frameworks between those related to ASF management and other regulatory areas, such as forest protection laws, animal welfare provisions, or biodiversity conservation. They brought up uncertainties regarding legal provisions such as restrictions on forest utilization, prohibitions on the use of certain materials like feed and bedding from restricted zones, and complexities around obtaining permissions for building fences, which can vary depending on land ownership and local regulations. It was found that there is currently a lack of clarity regarding the primary authority at the federal level, prompting questions about which entity holds responsibility, and which legal framework should prevail as definitive. For example, forestry stakeholders referred to a potential conflict between the duty to control forest pests, such as European spruce bark beetle *(Ips typographus)*, and ASF zone access restrictions during outbreaks.

#### 3.3.4 Challenges in communication and coordination

Communication challenges were identified between authorities, other stakeholders, and the public, coupled with information gaps and general uncertainties. Stakeholders emphasized the importance of clear role definitions and the coordination of measures across national and regional levels. Effective leadership from national and cantonal authorities, task forces for crisis management and preparative training sessions before the outbreak occurs were frequently mentioned.

#### 3.3.5 Animal welfare and environmental issues

Stakeholders expressed concerns regarding the welfare of domestic pigs, wild boars, and other wildlife affected by the planned control measures. Key issues included the impact of containment measures such as fences and hunting bans on animal welfare, the regulation of wildlife populations, and the broader (unpredictable) ecological effects on ecosystems and nature. Stakeholders emphasized that a proper scientific basis is required before using these potentially harmful measures.

## 4 Discussion

The study revealed a wide array of stakeholder groups of diverse professions, and varying roles, responsibilities, and interests related to ASF control in wild boar in Switzerland. The presence of such a broad spectrum of stakeholders highlights the interconnectedness and interdependence of many elements within a complex system that is typical of wildlife disease control (24). Such disease control must incorporate all stakeholders, ensuring that decision-making processes are as inclusive and adaptive as possible to accommodate diverse perspectives in response to evolving challenges (25).

### 4.1 Stakeholder analysis and categorization

We applied a participatory methodology for our study, which has previously been employed in similar ASF work. While Jori et al. (11) concluded on a stakeholder list similar to ours in Europe, some sub-categorizations of stakeholders were done differently. Additionally, we included stakeholders absent from Jori et al.’s list, such as the veterinary pharmaceutical industry, private forest owners, search dog units, and drone operators. These groups were included due to their relevance to key control activities, such as managing forested areas where wild boar are present in the case of private forest owners, and supporting carcass detection in the case of search dog units. The veterinary pharmaceutical industry plays a role in researching and potential distributing vaccines against ASF. Drone operators contribute to carcass detection and wild boar monitoring, for example in agricultural fields prior to harvest. Similarly, Hsu et al. (26) used participatory frameworks in the Philippines to combine expert and stakeholder perspectives, underscoring the universal value of collaborative strategies in designing effective, context-specific ASF control measures.

Effective ASF control relies on understanding stakeholder roles, influence, and engagement. Active collaborators are central to ASF control efforts. Regular task force meetings and coordinated planning are essential to ensure strategic alignment (20, 27–31). Strategic partners support implementation through resources, logistics, and enforcement, but require active engagement, such as involvement in policy development, due to limited interest (20, 27–31). Informed advocates can spread awareness and support inclusive policies but may also raise concerns about control measures (e.g., related to animal welfare or environmental impact), which can delay or complicate implementation. Regular consultations help address concerns and foster collaboration (20, 27–31). Peripheral observers may influence ASF control indirectly through behaviors that spread the virus. Targeted public education campaigns aimed at reducing risky practices such as bringing pork products from ASF-affected regions, improper disposal of food waste, or poor biosecurity practices associated with hunting tourism may help mitigate their impact (20, 27–31).

The “affecting vs. affected” mapping showed that many stakeholders influence ASF control, highlighting the need for collective action but also the risk of misalignment between stakeholder roles, perceived responsibilities, and their engagement in implementation. Many “affecting” actors were also “Movers,” showing commitment to ASF control. The hunting sector notably emerged as both an influencer and a key participant, confirming its pivotal role in wildlife disease control as seen in previous studies. For instance, Schulz et al. (32) focused on hunters in Germany and assessed the acceptability of a classical swine fever surveillance system for wild boar using participatory methods. Their findings showed that involving hunters in surveillance planning helps integrate their expertise, which is often overlooked. They also assessed existing surveillance strategies to improve their effectiveness and implementation. Similarly, Stončiūtė et al. (9) and Urner et al. (10) revealed that Lithuanian and Latvian hunters value trust in veterinary authorities but face challenges such as ethical concerns such as hunting female wild boar and insufficient financial or logistical incentives for passive surveillance. In our study, such concerns were also raised, particularly regarding animal welfare and economic risks. Nevertheless, Swiss hunters were categorized as “Movers” based on the power-interest mapping, recognizing the importance of ASF control and seeking greater involvement in decision-making. This categorization was developed by the research team and iteratively refined with expert input. This latter finding on resistance due to financial or ethical barriers contradicted our results suggesting that Swiss hunters are “Movers,” recognizing the importance of ASF control and seeking greater involvement in decision-making. Another aspect highlighted by the “affected vs. affecting” mapping was that many stakeholders in agriculture and forestry are primarily impacted by ASF measures but often excluded from planning. Their inclusion is crucial to protect interests, build trust, and prevent resistance. Compensation or involvement in decision-making can further support cooperation.

The “Movers, Floaters, and Blockers” framework further underscored the need for targeted stakeholder engagement. “Floaters” may shift positions as policies evolve. Transparent communication, participation, and economic incentives can help guide them toward constructive roles. “Blockers,” including animal protection NGOs and private forest users might oppose the planned ASF control due to concerns about animal welfare or perceived activity restrictions. In Italy, Palencia et al. (33) observed forest users disregarding ASF zone restrictions, as recorded by camera traps. Limited acceptance among forest users may lead to reduced compliance with control measures and interference with containment infrastructure. Addressing the forest users’ concerns requires demonstrating the broader benefits of ASF control, including ecosystem stability, agricultural protection, and economic security for farmers, the pig and pork industry, trade, and national economies. An adaptive, inclusive approach is essential to minimizing opposition against ASF control measures when these are needed.

The three mapping approaches used in this study provided complementary insights for ASF preparedness. The Power-Interest Grid helped identify which stakeholders should be engaged more closely based on their ability to influence ASF control and their level of involvement. The “affected vs. affecting” categorization clarified who implements measures and who is impacted by them, revealing where conflicts or trade-offs may arise. The “Movers, Floaters, and Blockers” model highlighted stakeholder attitudes and potential support or resistance. Taken together, these approaches offered a practical framework for identifying key actors, anticipating barriers, and informing inclusive and targeted decision-making.

### 4.2 Stakeholders’ concerns and consideration

Our study revealed stakeholder concerns ranging from personal economic impacts to collective challenges like coordination, legal frameworks, and bureaucracy. Altruistic concerns about environmental and animal welfare impacts also emerged, highlighting awareness of consequences beyond self-interest.

To ensure animal welfare, planning must occur before ASF introduction. This includes involving stakeholders (e.g., welfare groups, ecologists, hunters, and pig producers) in participatory processes and basing measures on scientific evidence. The feasibility and impact of population reduction methods like trapping and night shooting should be assessed in advance (11, 34). Once ASF occurs, rapid action is needed based on these contingency plans, and effective strategies must balance ecological, social, and economic factors and follow regulations. Hence, the current ASF crisis offers a chance to advance harmonized, evidence-based wildlife policy in Europe (35).

Governmental stakeholders emphasized coordination challenges, especially in Switzerland’s decentralized system. Standard protocols, digital tools, and national exercises could enhance clarity. ASF preparedness also requires broader training beyond veterinary services. Palencia et al. (34), based on expert insights from multiple countries and a literature review, note that while official veterinarians receive training, key administrative bodies (e.g., environmental authorities, the army) and external stakeholders (e.g., field veterinarians, hunters) are often excluded from training. They recommend joint field exercises such as carcass search, sampling, retrieval and disposal to strengthen logistics, along with specialized training in night shooting and trapping. In Italy, it became evident that communication with stakeholders about the severity of the ASF and the rationale behind interventions had been insufficient, drawing criticism from hunter and farmer associations in 2022 (34). The European Food Safety Authority has developed a comprehensive communication toolkit, including posters, stickers, and social media materials tailored to various stakeholders such as farmers, hunters, and veterinarians, available in multiple languages and ready to use by authorities (36). In addition, to improve coordination in federal systems, forming a strategic committee can be effective. Following the 2018 ASF outbreak in Belgium, such a committee comprising veterinary, wildlife, academic and administrative experts was key to successful elimination (37).

The stakeholders in our study also expressed concerns about control material shortages in the event of ASF incursion into Switzerland. These included items such as fencing equipment, materials for carcass recovery, and diagnostic sampling tools. Animal disease emergencies are known to be highly resource demanding and often exceed the typical capacities of most veterinary services (34). This could be overcome by centralized preparation and ongoing assessments of stockpiled materials and logistics hubs, coordinated resource management, and development of checklists addressing material and logistical preparedness.

Concerns about legal frameworks raised in our workshop also appear in other studies. For example, Migliore et al. (38) discussed the difficulties of applying ASF control under the EU Animal Health Law across multiple EU Member States, highlighting the need to coordinate veterinary, wildlife, and local regulations. While the EU faces challenges harmonizing laws between member states, Swiss stakeholders emphasized the complexity of working within a federal system, especially where cantonal and federal responsibilities differ. Brown et al. (39) reported similar issues in the fragmented governance system of the United States.

Economic concerns could be addressed through proactive financial measures, such as compensation schemes for affected stakeholders. Drawing inspiration from models in other ASF-affected countries, measures such as direct compensation for losses, compensation for hunters involved in carcass search or the culling of wild boars (34), compensation for forestry workers affected by restricted access and subsidies for preventive measures, could help to reduce the financial burden of ASF control on stakeholders. Rogoll et al. (40) also identified financial and logistical concerns by hunters and discussed financial incentives and reduced bureaucratic barriers as the most preferred strategies for increasing hunters’ participation, a dynamic that also resonated with Swiss stakeholders’ concerns.

Stakeholder discussions showed a collaborative attitude and no inter-institutional conflict. While trust into the governmental institutions was high, participants wished for clearer guidance. This contrasts with findings from other studies, such as those reporting Lithuanian hunters’ lack of trust in governmental institutions and their perception of insufficient cooperation with them (41). In crisis management, building stakeholder trust is key to effective response. Clear, structured, and transparently communicated contingency plans foster confidence by outlining challenges and strategies (42).

## 5 Strengths, limitations and conclusions of the study

One of the study’s primary strengths is the comprehensive participatory approach that engaged key stakeholders across the agriculture, hunting, forestry, and animal health sectors. Tools such as the Mendelow Power-Interest Grid and other mapping frameworks presented a structured approach to map stakeholders’ influence and interests, enabling a nuanced understanding of their perspectives.

Despite its strengths, the study has several limitations. Although having been able to compare our findings to those of other studies in Europe, we focused on the Swiss context, which limits the generalizability of findings beyond Switzerland. In addition, selection bias of participating stakeholders may have influenced findings, as participants were likely more supportive of ASF control measures, particularly in the hunting and pig sectors, where representatives aligned with “Mover” perspectives. While this limits variability of stakeholders’ perspectives, it provides valuable insights to capture the potential of interacting with actively engaged stakeholders. In addition, through the selection process of stakeholder groups’ representatives we believe that individuals with a common perception were selected in the focus group interviews. A potential limitation of the stakeholder mapping is that, although the categorization was iteratively refined based on expert interviews and shown to stakeholders during the focus group discussions, the final placements were not explicitly reviewed or confirmed by all stakeholders themselves. This may have introduced interpretation bias regarding perceived power and interest. Finally, interpretation is inherent in qualitative research; we followed established methodologies, such as reflexive thematic analysis, and repeated discussions of findings within the study team to improve rigor and trustworthiness of findings.

This study underscores the complexity of ASF management within Switzerland’s federated system, where coordination between federal and cantonal authorities is essential yet challenging. Resource shortages and legal ambiguities highlight the need for improved strategic planning and clearer regulations, including streamlined bureaucracy and better resource allocation. Our findings support Jori et al.’s (11) assertion that effective outbreak preparedness depends on early stakeholder engagement, public awareness, and comprehensive training, key steps that should be implemented before ASF is introduced. Our stakeholder analysis identifies key networks that can serve as access points for future participatory ASF projects. Ongoing stakeholder collaboration is crucial for refining ASF strategies and enhancing outbreak resilience. Future research should focus on concrete proposals to address stakeholder concerns through government actions and potential revisions to federal technical guidelines for their effective implementation.

Integrating participatory processes into ASF control can enhance Switzerland’s capacity to address this challenge effectively. This inclusive approach fosters trust, collaboration, and stakeholder engagement, shifting efforts toward a proactive and coordinated strategy.

## Data Availability

The original contributions presented in the study are included in the article/Supplementary material, further inquiries can be directed to the corresponding author.
