# Supplementary material for: Identifying stakeholders and analyzing their concerns about African swine fever control in wild boar
Source: Front Vet Sci. 2025 Jul 29;12:1602027. doi: 10.3389/fvets.2025.1602027 (PMC12341387; doi:10.3389/fvets.2025.1602027)
Supplement: Supplementary file 1 [file Data_Sheet_1.docx]

Supplementary Material

# Supplementary Figures and Tables

## Supplementary Figures


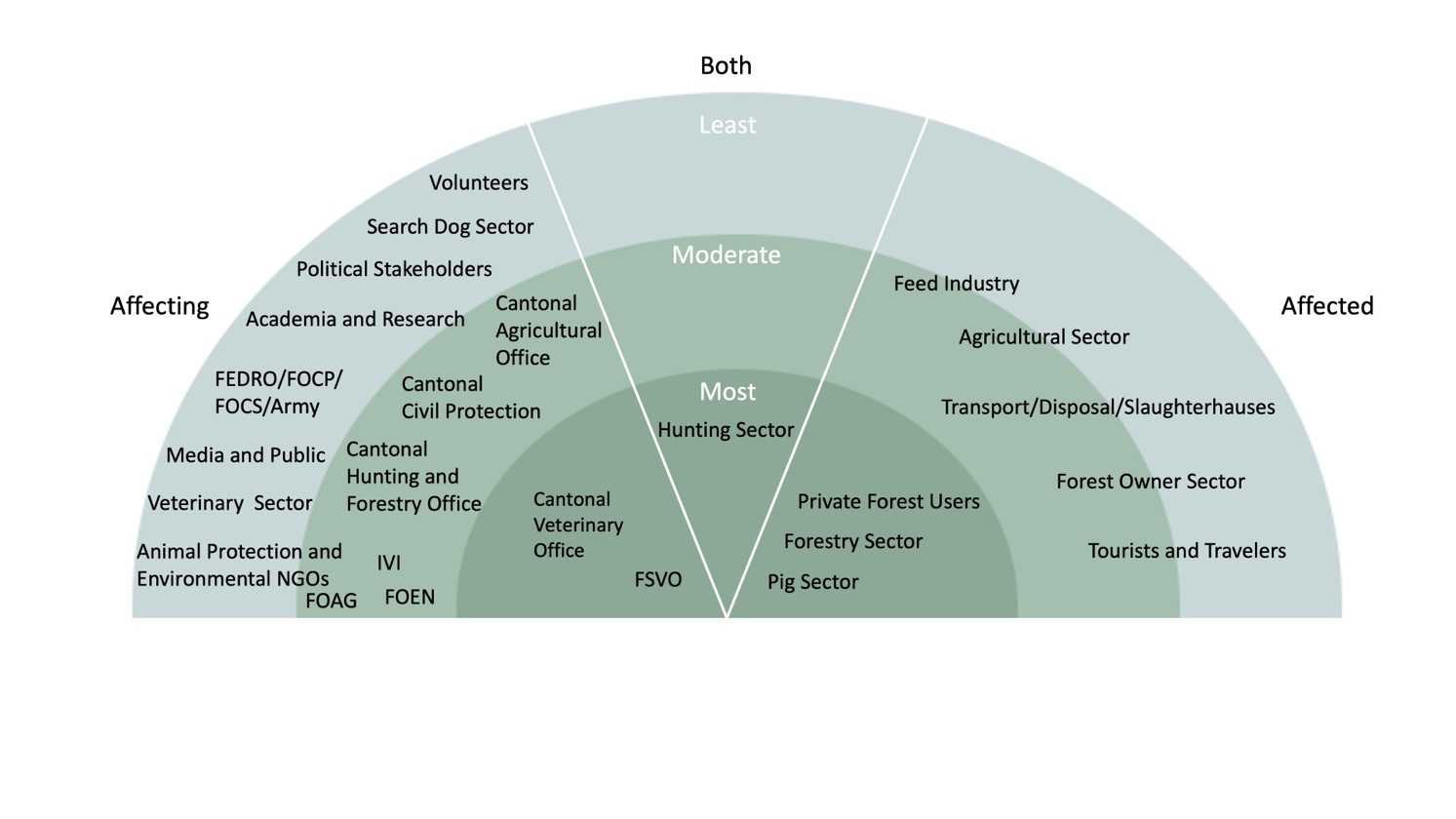


Supplementary Figure 1. “Affecting vs. Affected” Mapping Result: The figure illustrates the mapping of stakeholders involved in the control of African swine fever in wild boar in Switzerland. Stakeholders are categorized based on whether they are affected by or have an influence on the control of ASF in wild boar. Additionally, they were classified into different impact levels: least, moderate, or most (1). Abbreviations: FSVO: Federal Food Safety and Veterinary Office, FOEN: Federal Office for the Environment, IVI: Institute of Virology and Immunology, FOAG: Federal Office for Agriculture, FEDRO: Federal Roads Office, FOCP: Federal Office for Civil Protection, FOCS: Federal Office for Civilian Service.


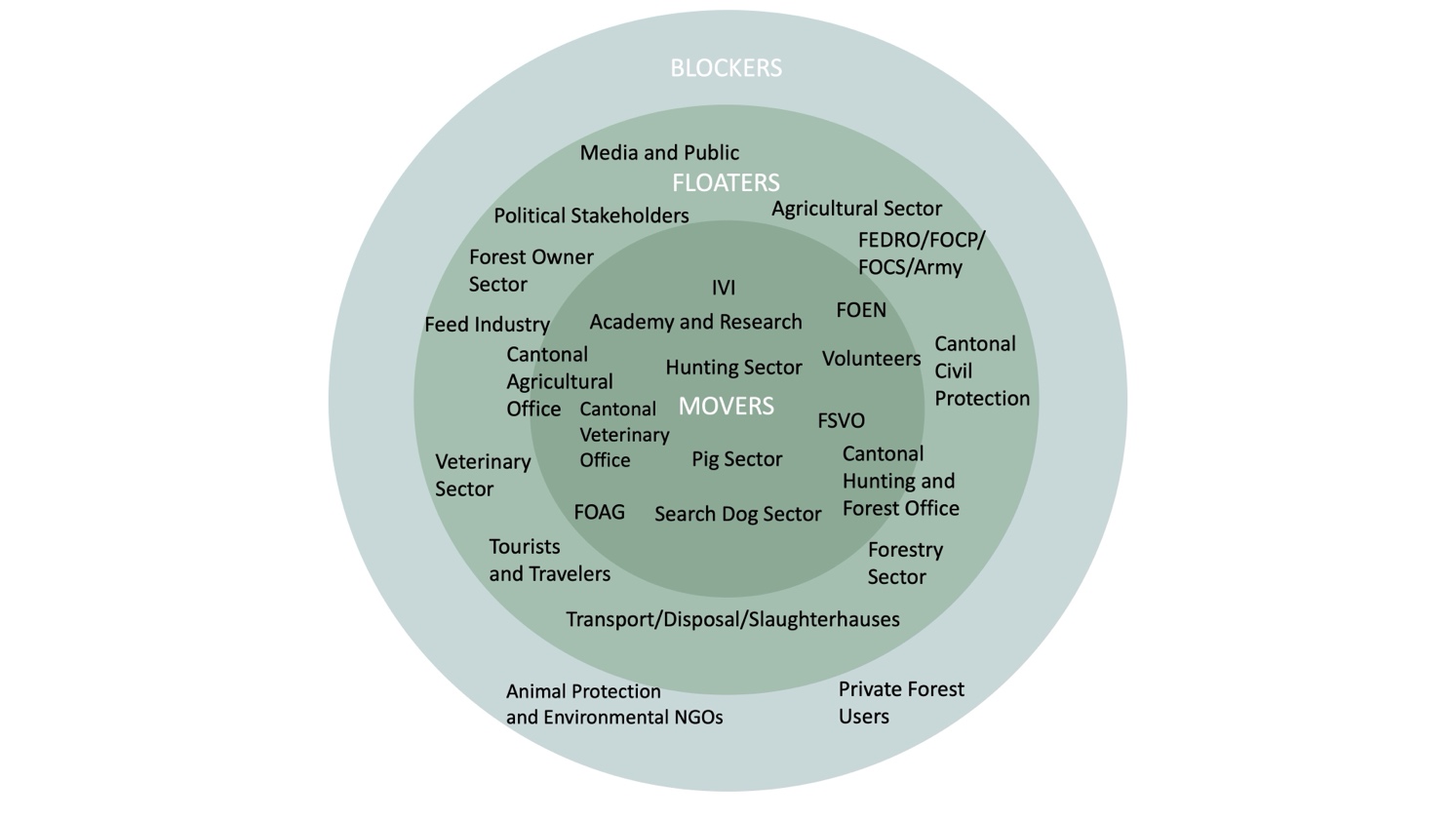


Supplementary Figure 2. “Movers vs. Floaters vs. Blockers” Mapping Result: The figure illustrates the mapping of stakeholders involved in the control of African swine fever in wild boar in Switzerland. Stakeholders are classified as movers, floaters, or blockers. Movers actively support and drive the implementation of control strategies, floaters remain neutral or adaptable, and blockers can oppose or hinder the measures due to various concerns or interests (2). Abbreviations: FSVO: Federal Food Safety and Veterinary Office, FOEN: Federal Office for the Environment, IVI: Institute of Virology and Immunology, FOAG: Federal Office for Agriculture, FEDRO: Federal Roads Office, FOCP: Federal Office for Civil Protection, FOCS: Federal Office for Civilian Service.

## Supplementary Tables

Supplementary Table 1. Stakeholder identification in alignment with the Reporting Items for Stakeholder Analysis (RISA) tool by L. Franco-Trigo et al. (3): The RISA tool provides a structured guideline to enhance the quality and transparency of stakeholder analysis reporting.

| **Clarifications** | **Information reported** |
| --- | --- |
| **Aim of the stakeholder analysis** | The objective was to systematically identify relevant stakeholders involved in ASF control in wild boar in Switzerland, assess their concerns, and categorize them based on their roles, interests, and influence. |
| **Reasons for conducting the stakeholder analysis** | The analysis was conducted to support ASF control planning by identifying key actors, their concerns, and their roles in decision-making, ensuring participatory research and strategic stakeholder engagement. |
| **System boundaries for the analysis** | Stakeholders were analyzed at the national and regional (cantonal) levels within Switzerland. |
| **Direction of the analysis (prospective vs retrospective)** | The analysis was prospective, designed to inform ASF preparedness and response strategies before an outbreak occurs. |
| **Individuals conducting the stakeholder analysis** | The research team consisted of an expert in veterinary public health and epidemiology, a doctoral student specializing in veterinary public health, as well as consultants with expertise in social sciences and public management. |
| **Data collection duration** | Data collection spanned from August 2023 to January 2024, including interviews and focus group discussions. |
| **Stakeholder definition applied for the analysis** | A stakeholder was defined as any person, group, or organization with an interest or concern in ASF control in wild boar, either being affected by or affecting control measures. |
| **Steps carried out/process followed for stakeholder identification** | Stakeholders were identified through desk research, expert interviews, and literature reviews, followed by validation in focus groups. |
| **Source of information for stakeholder identification** | Data was gathered from policy documents, scientific literature, expert interviews, and stakeholder consultations. |
| **Data collection methods for stakeholder identification** | Literature review, semi-structured expert interviews (n=11), and focus group discussions (n=4). |
| **Data display / Presentation of results for stakeholder identification** | Results were presented in tables, diagrams (e.g., Mendelow’s Grid), and narrative synthesis. |
| **Identification of stakeholders’ stakes/interests** | **Information reported** |
| **Steps carried out/process followed** | Stakeholder concerns were identified during interviews and workshops, then analyzed using reflexive thematic analysis. |
| **Source of information** | Stakeholders themselves via workshops. |
| **Data collection methods** | Qualitative data collected through interviews and focus groups, supported by document analysis. |
| **Data analysis** | Thematic coding framework (Reflexive thematic analysis) was applied to categorize concerns into five themes: animal welfare, economic risks, material shortages, legal challenges, and coordination/communication issues. |
| **Data display / Presentation of results** | Concerns were summarized in tables and figures, with key quotes included. |
| **Stakeholder differentiation/categorization or prioritization** | **Information reported** |
| **Stakeholder attributes and definitions** | Stakeholders were categorized based on power, interest, role (affecting vs. affected), and stance (Mover, Floater, Blocker). |
| **Steps carried out/process followed** | Attributes were derived from interviews and mapped using Mendelow’s Grid and additional classification frameworks. |
| **Source of information** | Interviews, focus groups, and policy documents. |
| **Data collection methods** | Qualitative interviews and structured discussions in focus groups. |
| **Data analysis** | Power-Interest Grid (Mendelow), Affecting vs. Affected framework, and Movers-Floaters-Blockers classification were used for analysis. |
| **Data display/Presentation of results** | Stakeholder categories were illustrated in tables and figures. |
| **Investigation of the relationships between stakeholders** | **Information reported** |
| **Steps carried out/process followed** | Stakeholder interactions were explored through workshops. |
| **Relationships analysed** | Communication gaps and collaboration structures were identified |
| **Timeframe for the relationships analysed** | Present and potential future collaborations were considered. |
| **Source of information** | Stakeholder perspectives gathered from interviews, focus groups, and workshops. |
| **Data collection methods** | Interviews, focus groups, and workshop exercises. |
| **Data analysis** | Stakeholder mapping |
| **Data display/Presentation of results** | Power vs. interest, affecting vs. affected, and Mover, Floater, Blocker diagram. |
| **Measures to ensure trustworthiness and reliability of stakeholder analysis** | **Information reported** |
| **Arguments that show why the results of the analyses are credible/valid, reliable, etc.** | Data triangulation (multiple sources), reflexivity in thematic coding, and stakeholder validation ensured rigor. |
| **How the results will be used; strategies for stakeholder engagement based on the results of the stakeholder analysis / how findings influenced the stakeholder engagement; recommendations for the future** | Results inform ASF control policies by emphasizing the need for coordinated federal-cantonal collaboration, targeted communication strategies, and improved legal frameworks. Stakeholder engagement strategies include participatory workshops and structured communication channels to maintain ongoing involvement. |

Supplementary Table 2. Stakeholder Concerns: Reflexive Thematic Analysis of Workshop Statements (Part 1): Initial coding of stakeholder statements from ASF control workshops, based on reflexive thematic analysis following Braun and Clarke (4). Each statement was assigned one or more codes capturing key concerns related to ASF control measures.

| **Stakeholder Statement** | **Code** |
| --- | --- |
| No worries, except for a lot of work that comes with the control measures. | Increased Workload |
| Proper local adaptation of control measures in terms of location and timing. Every place is different, and each principle must be implemented accordingly. | Local Differences and Adaptation |
| Whether (control measures) are effective and the fact that it takes a long time. Communication is very important. | Long Duration, Communication |
| Concern that we get lost in paperwork, miss the goal, and take the wrong turn because too many stakeholders are involved, potentially neutralizing each other. | Administrative Effort, Stakeholder Conflicts |
| The biggest concern is meat consumption. If slaughtering is no longer possible, it would not comply with animal welfare laws because pigs grow too large. How can people move around? And finally, financial losses. | Decline in Meat Consumption, Animal Welfare (Domestic Pigs), Financial Losses |
| Strong negative impact on other wild animals living in the forest and livestock. Methods may be used uncoordinatedly and might not be effective. Measures taken may not be respected by everyone. | Impact on Other Wild Animals, Animal Welfare (Domestic Pigs), Uncoordinated Efforts, Stakeholder Conflicts |
| Very complex and interdisciplinary problem → Will it work? Are we entering at the right point, and do we have the courage to escalate early? | Complex System, Many Stakeholders, Reaction Speed |
| Is everyone informed in time, and does the control work quickly enough? | Communication, Reaction Speed |
| Coordination among all parties. Condition and quantity of sample material. Sample flow, material shortages? | Coordination, Availability of Materials/Samples |
| AVET cannot implement necessary measures quickly enough. We know what needs to be done… but let's see what we can do. Fence construction. Perseverance at all levels? | Reaction Speed, Perseverance |
| How do we protect domestic pigs? Many are free-range. What should be done with the pigs? Will consumers still be interested in pork? Clear role distribution? Can we react quickly? Is the material (e.g., for fence construction) readily available? Do we have the authority to set up fences, etc.? | Animal Welfare (Domestic Pigs), Decline in Meat Consumption, Communication, Material Availability, Administrative Effort |
| Uncontrolled situation, the plan does not work. What surprises are coming our way? Surely, a large part of the population has never heard of this before. What happens when the general population has to face restrictions? Animal welfare. | General Uncertainty, Poor Public Information, Animal Welfare |
| What role does the hunting community play? Do they have the necessary knowledge? What happens if wildlife populations cannot be managed because hunting is banned? | Unclear Role Distribution in Hunting, Wildlife Welfare |
| Forest owners. What are the costs? | Forestry Finances |
| Federalism could be a problem → e.g., 'Crisis Task Force' → Which crisis task force? Cantonal? Federal? Who exactly? | Unclear Role Distribution, Federalism, Unclear Crisis Task Force |
| Logistics and preparation | Logistics, Preparation |
| The scale is unknown | Uncertainty |
| Bureaucracy | Bureaucracy |
| Time, it will take a long time | Long Duration, Perseverance |
| Use, restricted access to forest exploitation | Restrictions on Forest Use |
| Wild boar population will be significantly reduced due to an outbreak | Wild Boar Welfare |
| Hunting ban | Hunting Ban |
| Not sufficiently prepared | Insufficient Preparation |
| Time and workload | Increased Workload |
| The first case is detected too late | Reaction Speed, Late Response |
| Finances | Finances |
| Pig farm operators are uncertain. What happens to contract farming operations → delivery, livestock trading → what happens if they can no longer trade? | Uncertainty in Pig Farming Operations, Animal Welfare (Domestic Pigs) |
| Collection points for wild animal carcasses are not well prepared, no warm water, etc. | Insufficient Preparation |
| Are we fast enough with early detection? | Reaction Speed, Late Response |
| Farm operators are differently prepared → will we have enough material when needed? | Uncertainty in Pig Farming Operations, Material Shortages |
| What happens to bedding, feed → can we still use it? | Uncertainty in Pig Farming Operations, Lack of Information |
| Coordination—what happens at the farm level, daily business? | Animal Welfare (Domestic Pigs), Financial Concerns, Pig Farming Operations |
| Containment | Containment |
| Resources | Resources |
| Forestry workers banned from working | Forestry Work Ban |
| Wood utilization no longer possible | Financial Concerns for Foresters |
| Population control of roe deer and red deer | Animal Welfare, Wildlife Welfare |
| Forestry personnel being used as police → not their intended role… | External Control, Threat to Professional Practice |
| The right measures taken by the right people at the right time to prevent long delays. | Long Duration, Coordination, Effectiveness of Measures |
| The right knowledge about ASF (African Swine Fever) in the right place. | Information/Knowledge |
| Peacetime is not crisis time – difficult to be pragmatic during peacetime. | Uncertainty, Insecurity |
| What sample materials are available? Where are the dogs, etc.? Are all areas covered? | Resource Scarcity, Sufficient Preparation? |
| Information concept is also important (coordination); how do we reach target groups and through whom? Important that everyone receives the same communication; communication should be addressed in a timely manner because once it starts, it's too late! People should be made aware—what does it mean for the public? Surely, multi-level communication; does such a system exist? What is available and where? | Information Concept, Communication, Coordination, Early Information, Public Involvement, Information Sources |
| Is there enough diagnostics? Just a general overview of the situation: who can do what and where? From which pool does the carcass search personnel (deployment personnel) come? | Resource Scarcity, Resource Overview, Resource Planning, Resource Coordination, Availability of Personnel |
| Lead from the Federal Food Safety and Veterinary Office (BLV): Expected task force—who would be involved? People from affected cantons, who from which authority, etc.? | Federal Leadership, Organization Coordination, Task Force, Federalism |
| Finances: who pays for what? What is paid by the federal government, what by the cantons; which industry? Who buys materials? | Financial Organization, Financial Concerns, Resource Planning, Resource Procurement |
| Networking: Are all cantons involved? How does collaboration work? What about neighboring countries? | Stakeholder Networking, Federalism, Coordination, Organizational Collaboration |
| Population/Target Population: What is the actual goal for wild boars? Population size? | Wild Boar Regulation, Planning |
| Does the BLV have crisis management expertise? Does it have a crisis leadership team (task force)? | Federal Leadership, Task Force, Coordination, Leadership |
| Nationwide recording of available materials, manpower, drones, sniffer dogs, fence materials, etc.; purchase fence material internationally and create a national depot. | Resource Management, Resource Planning, Resource Overview, Funding Question |
| Legal basis for fence construction: Is it needed? On public land, it is relatively easy (except for nature conservation, etc.); on private forest land, it becomes relatively complicated! No building permit is required for mobile fences; in Italy, fences must be approved, which takes a long time. | Legal Requirements, Regulations and Approvals, Privatization |
| Harvest ban: Regulation in place; compensation? | Restrictions, Financial Concerns |
| Situation representation: where are the carcasses? Expansion to private individuals? | Public Involvement, Organizational Coordination, Digitalization |
| Animal welfare: Process of trap hunting/shooting—what does deployment with helicopters mean? | Wildlife Welfare, Impact on Wildlife, Ecosystem |
| What costs how much? Better assessment of certain measures. | Costs, Funding, Efficiency |
| When fences are set up in nature: Are there better and worse fences? What do fences mean for ecosystems and fauna? Are animal habitats fragmented? | Environmental Impact, Impact on Wildlife, Ecosystem, Scientific Basis |
| How long do restrictions such as fencing last? When a carcass is found: How long is the area classified as an ASF-infected area? Assessment by epidemiologists? | Duration of Restrictions, Time Planning, Ecosystem, Scientific Basis, Information, Uncertainty |
| Are legal frameworks in place? If not, are they necessary? Do they contradict each other? | Regulations and Laws |
| Who has the lead function at the federal level? Who represents the federal government (BLV, BAFU, BLW, etc.)? Which legislation is accepted, etc.? | Federal Leadership, Coordination, Leadership, Stakeholder Conflicts |
| Who is qualified to train others? Who can conduct training? For whom is it intended? Who pays, and where does it take place? | Training and Education, Organization, Qualification, Funding Question |
| How is the public informed? | Communication Concept, Public Involvement |
| Compensation for all affected groups? | Funding Question, Financial Concerns |
| Animal welfare? If outdoor access is closed in conventional pig farming: less space, animal welfare is no longer ensured, etc. What is more important: Animal Epidemic Law vs. Animal Welfare Law? | Animal Welfare (Domestic Pigs), Legal Conflicts, Regulations, Uncertainties |

Supplementary Table 3. Stakeholder Concerns: Reflexive Thematic Analysis of Workshop Statements (Part 2): Thematic organization of stakeholder concerns derived from workshop statements on ASF control. Codes from initial analysis were grouped into broader themes through an iterative, interpretive process based on Braun and Clarke’s reflexive thematic analysis (4).

| **Theme** | **Content** | **Codes** |
| --- | --- | --- |
| ****Animal Welfare and Environmental Issues**** | Welfare concerns for domestic pigs, wild boars, and other wildlife; impact of ASF control measures on ecosystems and nature; regulation of wildlife populations; effects of fences and hunting bans on animal welfare. | Animal Welfare (Domestic Pigs), Wildlife Welfare, Impact on Other Wild Animals, Hunting Ban, Environmental Impact, Ecosystem, Scientific Basis, Duration of Restrictions, Wild Boar Welfare. |
| ****Challenges in Communication and Coordination**** | Communication between authorities, stakeholders, and the public; information gaps and uncertainties; clear role definitions; coordination of measures across national and regional levels; leadership at federal and cantonal levels; crisis management by task forces; coordination of measures; training and qualifications. | Communication, Poor Public Information, Information/Knowledge, Coordination, Early Information, Public Involvement, Stakeholder Networking, Organizational Collaboration, Unclear Role Distribution in Hunting, Federal Leadership, Task Force, Training and Education. |
| ****Legal Frameworks and Bureaucratic Obstacles**** | Legal requirements, regulations, and their influence on ASF response and control measures; conflicts between different laws; bureaucratic hurdles. | Bureaucracy, Administrative Effort, Stakeholder Conflicts, Federalism, Legal Requirements, Regulations and Approvals, Privatization, Restrictions, Legal Conflicts, External Control, Threat to Professional Practice. |
| ****Material Shortages**** | Availability of materials and personnel; planning and provision of resources; logistical challenges in implementing measures; challenges in response speed and timely preparation. | Resource Scarcity, Availability of Materials/Samples, Resource Planning, Resource Coordination, Availability of Personnel, Resource Overview, Resource Management, Resource Procurement, Logistics, Preparation, Insufficient Preparation. |
| ****Economic Risk**** | Impact of ASF control measures on various economic sectors, particularly forestry and agriculture; financial concerns of affected stakeholders; issues of compensation and funding. | Financial Losses, Forestry Finances, Finances, Restrictions on Forest Use, Decline in Meat Consumption, Forestry Work Ban, Compensation for Affected Groups, Funding and Costs, Economic Concerns. |

1. Burgers, P., Iskandar, H., Bubungangkawijaya., Permana, R., and Farida, A. (2014). Landscapes and the voluntary carbon market, West Sumatra. ETFRN News, 56, 132–138.

2. Eguren, I.R. (2011). Theory of Change: A Thinking and Action Approach to Navigate in the Complexity of Social Change Processes. <https://www.academia.edu/10686540> [Accessed January 30, 2025].

3. Franco-Trigo L, Fernandez-Llimos F, Martínez-Martínez F, Benrimoj SI, Sabater-Hernández D. Stakeholder analysis in health innovation planning processes: A systematic scoping review. Health Policy. 2020;124(10):1083-1099. doi:10.1016/j.healthpol.2020.06.012

4. Braun, V., and Clarke, V. (2006). Using thematic analysis in psychology. Qualitative Research in Psychology, 3(2), 77–101. doi:10.1191/1478088706qp063oa
